# Removal of Radioactive Iodine Using Silver/Iron Oxide Composite Nanoadsorbents

**DOI:** 10.3390/nano11030588

**Published:** 2021-02-26

**Authors:** Mah Rukh Zia, Muhammad Asim Raza, Sang Hyun Park, Naseem Irfan, Rizwan Ahmed, Jung Eun Park, Jongho Jeon, Sajid Mushtaq

**Affiliations:** 1 Department of Nuclear Engineering, Pakistan Institute of Engineering and Applied Sciences, P. O. Nilore, Islamabad 45650, Pakistan; Mahrukhzia14@gmail.com (M.R.Z.); naseem@pieas.edu.pk (N.I.); Rizwanahmed@pieas.edu.pk (R.A.); 2 Advanced Radiation Technology Institute, Korea Atomic Energy Research Institute, Jeongeup 56212, Korea; masimraza@ust.ac.kr (M.A.R.); parksh@kaeri.re.kr (S.H.P.); 3 Radiation Science and Technology, University of Science and Technology, Daejeon 34113, Korea; 4 Department of Applied Chemistry, College of Engineering, Kyungpook National University, Daegu 41566, Korea; pje1204@knu.ac.kr

**Keywords:** adsorbents, radioactive wastes, radioactive iodine, desalination, nanocomposites

## Abstract

Efficient and cost-effective removal of radioactive iodine (radioiodine) from radioactive contaminated water has become a crucial task, following nuclear power plant disasters. Several materials for removing radioiodine have been reported in the literature. However, most of these materials exhibit some limitations, such as high production cost, slow adsorption kinetics, and poor adsorption capacity. Herein, we present silver/iron oxide nanocomposites (Ag/Fe_3_O_4_) for the efficient and specific removal of iodine anions from contaminated water. The Ag/Fe_3_O_4_ were synthesized using a modified method and characterized via scanning electron microscopy, transmission electron microscopy, and X-ray diffraction analyses. This adsorbent showed a high adsorption capacity for iodine anions (847 mg/g of the adsorbent) in pure water. Next, Ag/Fe_3_O_4_ was applied to the removal of radioiodine, and high removal efficiencies were observed in water. In addition, its desalination capacity was retained in the presence of competitive ions and varied pH. After the adsorption process, Ag/Fe_3_O_4_ was easily removed from the water by applying an external magnetic field. Moreover, the same operation can be repeated several times without a significant decrease in the performance of Ag/Fe_3_O_4_. Therefore, it is expected that the findings presented in this study will offer a new method for desalinating radioiodine in various aqueous media.

## 1. Introduction

The safe and reliable treatment of radioactive waste is inevitably linked to the safe production of nuclear energy [1,2]. Environmental damage caused by radioactive waste has attracted global attention. Radioisotopes, such as radioactive iodine (radioiodine), which exhibit a high degree of dispersion in water and air, are produced by nuclear fission. These can exert long-term adverse effects on human lives [3,4,5]. Notably, the global concern regarding nuclear waste leakage was kindled by the Fukushima accident in 2011 [6]. Further, the Chernobyl disaster in Ukraine occurred in 1986 wherein iodine radioisotopes were a major component of the radioactive contamination [7]. Moreover, the continuous operation of nuclear power plants can produce and introduce trace amounts of radioiodine into the environment [8]. Radioiodine has also been extensively used in the diagnosis of diseases and treatment of thyroid cancer on the basis of the selective uptake of iodine into the thyroid [9,10]. Consequently, the introduction of trace amounts of radioiodine from nuclear medicine research institutes also needs to be considered. For example, the medical applications of iodine-131 (^131^I; half-life: 8 days) and iodine-129 (^129^I; half-life: 15.7 × 10^6^ years) are considered to be the main generators of radioiodine waste [11,12,13]. The short-lived as well as long-lived radioisotopes of iodine can accumulate and cause serious damage to the human body. Therefore, the efficient treatment of radioactive iodine in nuclear wastes and contaminated water is an essential area of research. In past decades, various adsorbents such as graphene-based sorbents [14,15], deep eutectic solvents [16,17], hydrogelators [18], nanoporous carbons [19,20], polyacrylonitrile–chalcogel [21], microporous polymers [22,23,24], metal–organic frameworks (MOFs) [25,26], and functionalized zeolites [27,28] were employed to remove radioiodine that was dissolved in solutions and/or gaseous radioiodine. However, these materials exhibited several drawbacks, including low removal efficiency, slow adsorption kinetics, and high production cost. Furthermore, layered bismuth–iodine–oxide [29], titanate nanolamina [30], Mg–Al(NO_3_) layered double hydroxide (LDH) [31], and magnetite composites [32,33] have been employed to remove iodine. However, setbacks ranging from their poor reusability to their low adsorption capacities have limited the application of these methods. In previous studies, we reported that gold nanoparticles (AuNPs) immobilized adsorbents for the removal of radioiodine anions in aqueous media [34,35,36,37]. The method exhibited efficient and ion-selective desalination; however, the high cost of AuNPs-based systems hampered their large-scale syntheses and remediation applications.

**Figure 1:**
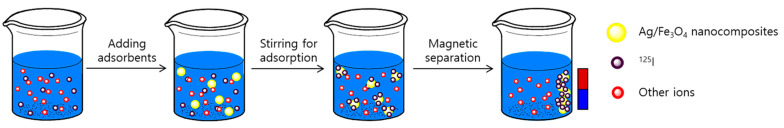
Schematic illustration of the desalination process using Ag/Fe_3_O_4_ composite nanoadsorbents.

Silver-based materials have also demonstrated a great potential for removing iodine owing to the high affinity of iodine toward silver [3,38,39]. In a typical procedure in the previous studies, silver nanoparticles or silver-based composite materials were immersed in the contaminated water to remove radioiodine. Thereafter, radioiodine containing solid waste was separated from the water via filtration or centrifugation. However, most of these methods require further steps to separate solid radioactive wastes from water after the desalination procedure. Moreover, the separation of nanosized adsorbents via these methods is time-consuming and non-applicable at an industrial scale. Thus, the development of additional cost-effective, efficient remediation procedures for radioactive wastes is still desired. Here, we designed a stable and efficient silver/iron oxide (Ag/Fe_3_O_4_) nanocomposite-based desalination system for the efficient removal of radioiodine from water. Compared with nonmagnetic silver composites, the magnetic nature of the Ag/Fe_3_O_4_ adsorbent is advantageous as it can be harvested by an external magnetic field. Therefore, the desalination procedure and recovery of radioisotope-containing adsorbents from treated water can be easy, rapid, and cost-effective (Figure 1).

## 2. Experimental Procedures

### 2.1. Materials

Radioiodine ([^125^I]NaI) was supplied by PerkinElmer in an aqueous sodium hydroxide (NaOH) solution. All the chemicals, such as iron(III)chloride hexahydrate (FeCl_3_·6H_2_O), iron(II)chloride tetrahydrate (FeCl_2_·4H_2_O), silver nitrate (AgNO_3_), NaOH, ethanol (C_2_H_5_OH), (3-aminopropyl)trimethoxysilane (H_2_N(CH_2_)_3_Si(OCH_3_)_3_, APTMS), hydroxylamine hydrochloride (NH_2_OH·HCl), sodium iodide (NaI), hydrochloric acid (HCl), sodium chloride (NaCl), sodium bromide (NaBr), sodium fluoride (NaF), sodium chlorate (NaClO_3_), sodium bromate (NaBrCO_3_), and potassium iodide (KI), were purchased from Sigma Aldrich Korea and utilized without further purification. The radioiodine removal experiments were performed using a radio-thin-layer chromatograph (TLC, AR-2000, Bioscan, USA) that was equipped with a dose calibrator (CRC-25PET) or automatic gamma counter (2480 automatic gamma counter, PerkinElmer, UK)Further, the nonradioactive iodine removal experiments were performed via ultraviolet–visible (UV–Vis) spectroscopy (UV–Vis spectrophotometer, Evolution™ 201/220, Thermo Scientific™, USA). The Fe_3_O_4_ and Ag/Fe_3_O_4_ composite nanoparticles were characterized via transmission electron microscopy (TEM; H-7650, Hitachi, Japan), field-emission scanning electron microscopy (FE-SEM; FEI Verios 460L, Philips, USA), and X-ray diffraction (XRD, Bruker, D2 PHASER). Magnetization measurements of nanomaterials were performed by a vibrating sample magnetometer (VSM JDM-13, Lake Shore, USA) at room temperature.

### 2.2. Synthesis of the Fe_3_O_4_ Nanoparticles

The Fe_3_O_4_ nanoparticles were prepared using a slightly modified, coprecipitation method [38]. Briefly, FeCl_2_·4H_2_O (1.99 g, 0.01 mol) and FeCl_3_·6H_2_O (5.41 g, 0.02 mol) were dissolved in water and sonicated for 30 min. The FeCl_2_·4H_2_O and FeCl_3_·6H_2_O solutions were mixed and placed in a three-neck bottle. The resulting solution was heated at 90 °C in a nitrogen stream, after which it was vigorously mixed in a deoxygenated atmosphere. Next, the aqueous NaOH solution was added dropwise, and the Fe_3_O_4_ nanoparticles were obtained as dark-brown precipitates. The precipitated mixture was stirred for an additional 6 h to achieve complete conversion. The Fe_3_O_4_ nanoparticles were extracted from the solution by applying an external magnetic field, washed several times with water and ethanol, and vacuum-dried for 2 h at 80 °C.

### 2.3. Synthesis of the Ag/Fe_3_O_4_ Composite Nanoadsorbents

The Ag/Fe_3_O_4_ composite materials were prepared via APTMS [40]. Briefly, the Fe_3_O_4_ nanoparticles (0.30 g) were dispersed in 150 mL of ethanol and sonicated for 45 min. Next, APTMS (1 mL) was injected into the reaction mixture and stirred for 10 h. The APTMS-functionalized Fe_3_O_4_ nanoparticles were extracted by applying an external magnetic field, washed several times with ethanol, and vacuum-dried for 2 h at 80 °C. Further, the APTMS-functionalized Fe_3_O_4_ nanoparticles (0.25 g) were dispersed in 100 mL of water, after which AgNO_3_ (0.30 wt.%) was added to the reaction mixture, followed by sonication for 1 h. For the preparation of the silver nanoparticles, NaOH (50 mL, 0.1 M) and hydroxylamine hydrochloride (45 mL, 0.05 M) were added to the reaction mixture and stirred for an additional 2 h. Finally, the Ag/Fe_3_O_4_ composites were extracted by applying a magnetic field, washed several times with water and ethanol, and vacuum-dried at 80 °C.

### 2.4. Adsorption of Nonradioactive Iodine(^127^I^−^) Using Ag/Fe_3_O_4_ Composite Nanoadsorbents

Adsorption efficiency of the Ag/Fe_3_O_4_ nanocomposites was determined by measuring the absorbance variation of nonradioactive NaI/KI via UV–Vis spectroscopy at a maximum wavelength, λ_max_ = 225 nm. Briefly, 100 ppm stock solution was prepared by dissolving KI in water, and the pH was maintained at 7. Further, the desired low concentrations were prepared by diluting the stock solution. In the adsorption experiment, the adsorbent, the Ag/Fe_3_O_4_ composites, were shaken with an aqueous KI solution of a given concentration at a different time interval. After the experiment, the adsorbent was removed by an external magnet. The iodine concentration of the treated solution was measured via UV–Vis spectroscopy.

The percentage removal efficiency of Ag/Fe_3_O_4_ nanocomposites was measured using Equation (1):

(1) $$\mathrm{Removal}\;\mathrm{efficiency}\;(\%)\;=\;\frac{\left(C_{0}-\;C_{e}\right)}{C_{0}}\;\times \;100$$

Equilibrium adsorption capacity of Ag/Fe_3_O_4_ nanocomposites, Q_e_ (mg/g), was determined using Equation (2):

(2) $$Q_{e}\;=\;\frac{\left(C_{0}-{\;C}_{e}\right)}{M}\;\times \;V$$

where Q_e_ (mg/g) is the quantity of I^−^ that was adsorbed on the adsorbent at equilibrium time, C_0_ (mg/L) is the initial concentration of I^−^ in the aqueous solution, C_e_ (mg/L) is the final concentration of I^−^ in the aqueous solution at time t, V (L) is the volume of the solution, and M (g) represents the mass of the adsorbents (Ag/Fe_3_O_4_).

### 2.5. Determination of Removal Efficiency in the Presence of Competitive Ions

The removal efficiency of Ag/Fe_3_O_4_ nanocomposites was investigated in the presence of competitive ions. Radioiodine, [^125^I]NaI (150 µCi), was diluted in an aqueous solution of NaCl, NaBr, NaF, NaClO_3_, NaBrCO_3_, or nonradioactive NaI (10 mL, 1.0 M). The Ag/Fe_3_O_4_ nanoparticles were stirred with the [^125^I]NaI solution of given radioactivity for 60 min. Next, the adsorbent was removed by an external magnet. The radioactivities of the supernatant and adsorbent material were measured using the radio-TLC system or dose calibrator.

### 2.6. Removal Efficiency in Different Aqueous Media

To investigate the removal efficiency of the adsorbents, [^125^I]NaI (150 µCi) was diluted in 10 mL of different aqueous media (pure water, 1× PBS, water at 80 °C, river water, 0.1 M NaOH, 0.1 M HCl, or 1.0 M NaI). Ag/Fe_3_O_4_ nanocomposites were stirred with the [^125^I]NaI solution of given radioactivity for 60 min. Subsequently, the adsorbent was removed by an external magnet. The radioactivities of the supernatant and adsorbent materials were measured using the radio-TLC system or dose calibrator.

### 2.7. Reusability of the Composite Nanoadsorbents

To investigate the reusability of Ag/Fe_3_O_4_ nanocomposites, [^125^I]NaI (150 µCi) was diluted in 10 mL of pure water. Ag/Fe_3_O_4_ (10 mg) was shaken with the [^125^I]NaI solution of given radioactivity for 60 min. Subsequently, the adsorbent was removed by an external magnet. The radioactivity in the supernatant and adsorbent materials was measured using a gamma counter. The experiment was repeated for up to seven cycles.

### 2.8. Adsorption Isotherm Studies

The adsorption isotherm was measured using the KI solution at an ambient temperature and neutral pH. Briefly, 5 mg of the Ag/Fe_3_O_4_ nanoparticles was treated with 100 mL of KI at different initial concentrations (100–200 ppm) with a constant increment of 10 ppm. The final concentration of iodine after adsorption procedure was determined via UV–Vis spectroscopy at different intervals. The adsorption of I^−^ (*Q_e_*) was calculated using Equation (2). The Langmuir and Freundlich isotherm models were applied to describe the equilibrium adsorption:

(3) $$\mathrm{Langmuir}\;\mathrm{equation}:\;\frac{C_{e}}{Q_{e}}=\;\frac{C_{e}}{Q_{max}}\;+\;\frac{1}{Q_{max\;}K_{L}}$$

(4) $$\mathrm{Freundlich}\;\mathrm{equation}:\;lnQ_{e}=\;lnK_{F}\;+\;\frac{1}{n}lnC_{e}$$

where C_e_ (mg/L) are concentrations of I^−^ at the initial and equilibrium times, respectively. Q_e_ (mg/g) is the quantity of I^−^ that was adsorbed on the adsorbing medium at the equilibrium time, and Q_max_ (mg/g) is the maximum adsorption capacity of the adsorbent. K_L_ and K_F_ are the Langmuir and Freundlich adsorption constants, respectively.

### 2.9. Adsorption Kinetics of I^−^ on the Adsorbents

The adsorption kinetics of I^−^ on Ag/Fe_3_O_4_ nanoparticles was determined using 100 ppm KI at pH 7 and room temperature. Briefly, 100 mL of KI (100 ppm) solution was shaken with 5 mg of Ag/Fe_3_O_4_ nanocomposites. At different times, the absorbent was separated from the solution by applying an external magnet and the concentration of I^−^ was determined via UV–Vis spectroscopy by measuring the absorbance variation at the maximum wavelength, λ_max_ = 225 nm. The adsorption capacity was fitted into the pseudo-first-order (PFO) and pseudo-second-order (PSO) kinetics equations with respect to time, as expressed in Equations (3) and (4), respectively.

(5) $$\mathrm{PFO}\;\mathrm{kinetic}\;\mathrm{model}:\;\ln\left(Q_{e}-Q_{t}\right)={\;\mathrm{lnQ}}_{e}-\;\frac{k_{1}t}{2.303}$$

(6) $$\mathrm{PSO}\;\mathrm{kinetic}\;\mathrm{model}:\;\frac{t}{Q_{t}}=\;\frac{1}{k_{2}Q_{e}^{2}}+\;\frac{t}{Q_{e}}$$

where Q_e_ and Q_t_ are the quantities of I^−^ (mg/g) at equilibrium and time t, respectively. The Fe_3_O_4_ nanoparticles were used in the control experiment under similar conditions. k_1_ (min^−1^) and k_2_ (g mg^−1^ min^−1^) are the PFO and PSO adsorption rate constants, respectively.

## 3. Results and Discussion

The adsorbent, Ag/Fe_3_O_4_ nanoparticles, were synthesized in two steps using a modified procedure, as shown in Figure S1a. To prepare Fe_3_O_4_ nanoparticles, a mixture of FeCl_2_·4H_2_O and FeCl_3_·6H_2_O was treated with NaOH at 90 °C, and the product was washed several times with water and ethanol, after which it was dried at a high temperature. In the next step, APTMS was coated on the surface of the Fe_3_O_4_ nanoparticles. Further, the silver layer was formed using AgNO_3_ in the presence of hydroxylamine hydrochloride and a base.

**Figure 2:**
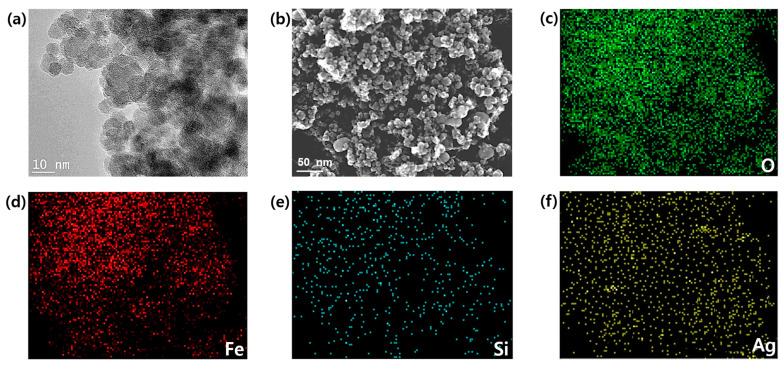
(**a**) TEM images of the Ag/Fe_3_O_4_ composite nanoparticles, (**b**) SEM image of the Ag/Fe_3_O_4_ composite nanoparticles, (**c**–**f**) EDS elemental mapping patterns of the Ag/Fe_3_O_4_ nanocomposites from (**b**).

**Figure 3:**
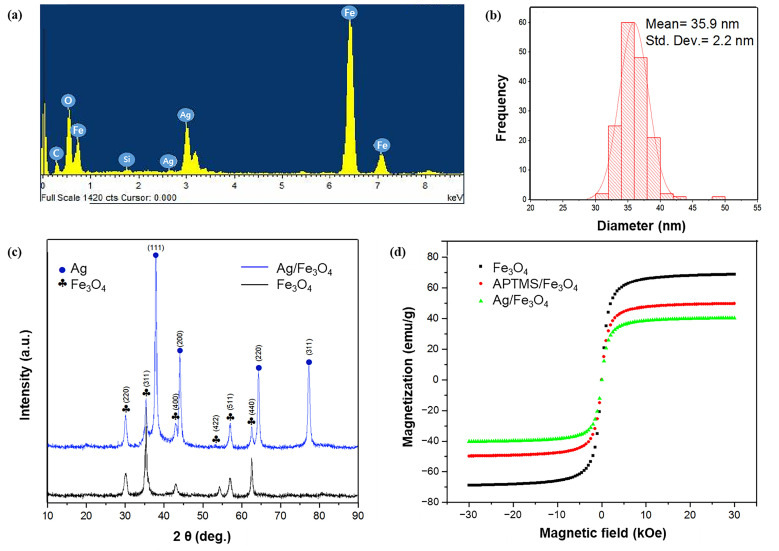
Characterization of the nanomaterials. (**a**) EDS spectrum of the Ag/Fe_3_O_4_ nanocomposites; (**b**) size-distribution histogram of the Ag/Fe_3_O_4_ with a standard deviation of 2.2 nm; (**c**) XRD analysis of the Fe_3_O_4_ and Ag/Fe_3_O_4_; (**d**) magnetic hysteresis loops of Fe_3_O_4_, APTMS/Fe_3_O_4_, and Ag/Fe_3_O_4_ nanocomposites at room temperature.

The particle morphology and size of the prepared nanoparticles were observed via SEM and TEM, respectively. SEM images of the bare Fe_3_O_4_ exhibited the nearly spherical shapes of the particles (Figure S2a,b), and a significant uniform particle-size distribution was observed. The observed average size of the Fe_3_O_4_ particle was ~27 nm (Figure S2c–e). The crystal structure and phase of the prepared nanoparticles were determined via X-ray diffraction (XRD). The strong Bragg peaks of (220), (311), (400), (422), (511), and (440) corresponded to the diffractions from the inverse spinel structure of Fe_3_O_4_ (Figure S2f). The energy-dispersive X-ray spectroscopy (EDS) analysis of the Fe_3_O_4_ nanoparticles revealed a set of peaks, which corresponded to iron as well as oxygen (Figure S3). The characterization data of Ag/Fe_3_O_4_ are shown in Figure 2. The TEM and SEM images of Ag/Fe_3_O_4_ nanocomposites showed the nearly spherical-shaped agglomerates, respectively (Figure 2a,b). The additional TEM data are shown in Figure S4. The elemental mapping patterns revealed the presence of the main elements, including O, Fe, Si, and Ag (Figure 2c–f). A new peak, which corresponded to silver, is evident compared with the Fe_3_O_4_ nanoparticles, thus confirming the presence of the silver coating on the Fe_3_O_4_ nanoparticles. In addition, the EDS data of the Ag/Fe_3_O_4_ nanoparticles revealed the presence of Fe, O, C, Si, and Ag atoms (Figure 3a). The presence of Si was observed after the surface modification of the Fe_3_O_4_ particles via APTMS. The observed particle size was 35.9 nm with a standard deviation of 2.2 nm (Figure 3b). The size of nanomaterials was further analyzed by the Scherrer equation (Tables S1 and S2). The XRD peaks revealed the crystalline nature of the nanoparticles. The peaks indicated the ultrafine nature and small crystallite size of the nanoparticles. The strong Bragg peaks of (111), (200), (220), and (311) corresponded to the diffractions from the FCC (Face-centered Cubic) structure of silver nanoparticles. The absence of spurious diffractions indicated the absence of significant impurities in the sample (Figure 3c). The magnetic properties of the nanoadsorbent were assessed by applying a magnetic field in the range of −30,000 to 30,000 Oe via vibrational sample magnetometry. The bare Fe_3_O_4_ nanoparticles exhibited the highest saturation magnetization (*M_s_*) value (67.84 emu/g). However, the value decreased to 49.48 and 40.34 emu/g because of the APTMS coating and combination of silver with the APTMS coating, respectively (Figure 3d). The surface modification and formation of the silver nanoparticles on the surface of the magnetite nanoparticles caused a decrease in the *M_s_* value. This result can be attributed to the presence of more diamagnetic material per gram of the material. Zero remanence and coercivity of the magnetization curve suggested that the nanoadsorbents possessed superparamagnetic properties. As shown in Figure S1c–e, Ag/Fe_3_O_4_ responded immediately to the external magnetic field, and the collected particles could be dispersed again by gentle shaking after removing the magnet. This result indicates that the adsorbents can be easily removed from wastewater via a simple separation procedure.

**Figure 4:**
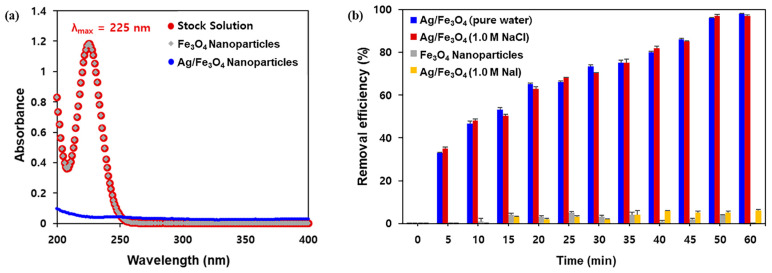
(**a**) UV absorption spectra of iodine after treatment with Fe_3_O_4_ or Ag/Fe_3_O_4_ composite nanoparticles in water; (**b**) removal efficiency of Ag/Fe_3_O_4_ nanocomposites at high salt concentrations, and the control study using Fe_3_O_4_ nanoparticles.

To perform the iodine adsorption experiments, nonradioactive iodide anion (I^−^) or radioiodine (^125^I^−^) were used. The calibration curve was plotted to determine the concentrations of I^−^ (λ_max_ = 225 nm) in aqueous media (Figure S5) via UV–Vis spectroscopy. First, the adsorbent was added to a 100 ppm KI solution in pure water or a NaCl solution, after which the amount of I^−^ absorbed on Ag/Fe_3_O_4_ was determined by comparing the UV absorbance at 225 nm for 1 h. Figure 4a reveals that I^−^ in aqueous media could not be captured by the unmodified Fe_3_O_4_. Conversely, silver-coated adsorbents efficiently removed I^−^ with a removal efficiency of ~100% in 1 h (Figure 4b). Interestingly, the excellent removal efficiency was also observed using 1.0 M NaCl solution with a Cl^−^ to I^−^ anion ratio ([Cl^−^]:[I^−^]) exceeding 10^3^:1, thereby indicating the ion-selective adsorption performance of the silver layer.

**Figure 5:**
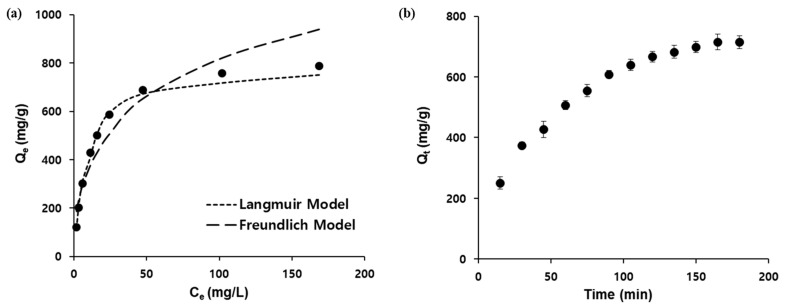
(**a**) Adsorption isotherm study employing the Langmuir and Freundlich models, (**b**) absorption kinetics as a function of time.

Linear fitting of the observed data according to the Langmuir (3) and Freundlich isotherm models (4) revealed that the adsorption mechanism of Ag/Fe_3_O_4_ was better described by the Langmuir equation with a correlation factor (*R*^2^) of 0.995 (Figure 5a). This result also indicated the monolayer adsorption mechanism, and the observed maximum adsorption capacity (*Q_max_*) obtained using Equation (3) was 847 mg/g. The corresponding parameters for these models are summarized in Table 1. The kinetic parameters of the adsorption are also pivotal to the practical application of the nanoadsorbent. The removal efficiency of I^−^ was measured as a function of time (5–180 min) to determine the optimum time for the desalination experiments (Figure 5b). The adsorption of I^−^ was rapid in the first 60 min, after which it became slower, before finally reaching a plateau after 180 min. The fitting results of the PFO and PSO kinetic models are shown in Figure S6a,b and Table 2. Based on the calculated kinetic parameters, it is clear that the PSO kinetic model fitted better with the kinetic results.

**Figure 6:**
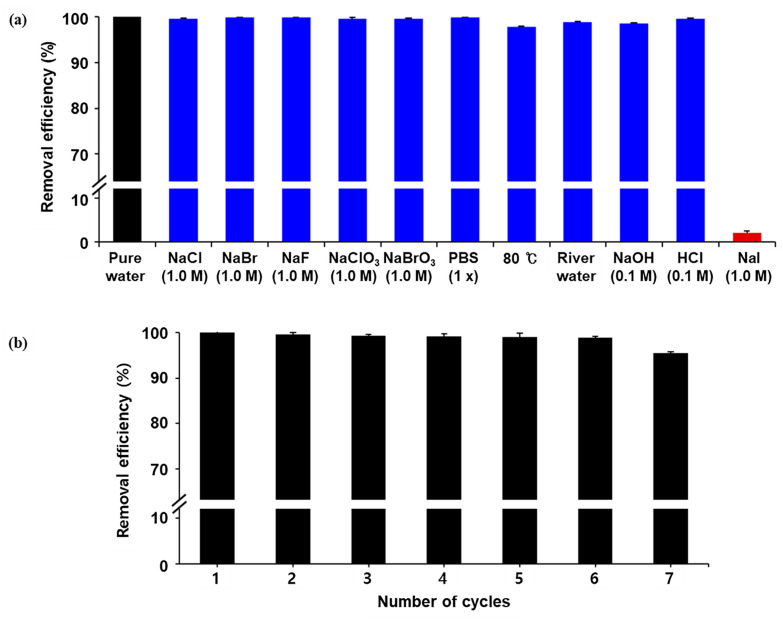
(**a**) Removal efficiency of the Ag/Fe_3_O_4_ nanocomposites in the presence of competitive ions, different aqueous solutions, and different pH conditions using radioiodine (^125^I); (**b**) removal efficiency of the Ag/Fe_3_O_4_ nanocomposites using radioiodine (^125^I) as a function of the number cycles.

Using these results, we investigated the desalination of radioiodine by Ag/Fe_3_O_4_. For this study, the same adsorbents were immerged into aqueous solutions containing 150 µCi of [^125^I]NaI. Afterward, the adsorbents were collected by an external magnet. The removal efficiency was determined by measuring the residual radioactivities in the solution and Ag/Fe_3_O_4_ nanocomposite. As shown in Figure 6a, high removal efficiencies were observed in the presence of other competing anions as well (e.g., Cl^−^, Br^−^, and phosphate). By contrast, the adsorption of radioiodine was completely inhibited in an aqueous solution of nonradioactive NaI, which might be due to the surface area of the adsorbents getting covered by an excess amount of I^−^. The desalination performance was also evaluated in other environments, such as varied pH values, river waters, and elevated temperatures. More than 99% of the radioactive iodines were captured by Ag/Fe_3_O_4_ in 1 h in these environments as well. These results demonstrated that Ag/Fe_3_O_4_ successfully and selectively captures I^−^ in the presence of mixed ion species. Next, the reusability of Ag/Fe_3_O_4_ was explored via the repetitive adsorption of radioiodine (150 µCi) from water. As shown in Figure 6b, a high removal efficiency (>99%) was observed in seven consecutive processes, suggesting that the adsorbent retained its stability. Moreover, the adsorbed radioiodine anions on the adsorbents were not readily released during the repeated operations.

Several studies have described various silver metal or silver oxide composite nanomaterials that can remove radioactive iodines [3,41]. Removal of unsettled adsorbents after water treatment requires further separation processes. Compared with the previous studies, this method offers a simpler and more efficient method for capturing radioactive materials from different aqueous solutions. Through a single operation for 1 h, most of the radioactive components were selectively captured by Ag/Fe_3_O_4_. Thereafter, the magnetic separation successfully recovered the I^−^-loaded adsorbent from the aqueous solvent. Additionally, the observed Q_max_ value was favorable compared with those obtained in previous studies (Table S3). As a result of the large-scale synthesis and characterization of Fe_3_O_4_ as well as the establishment of the formation of the silver layer, a large number of adsorbents that were used in this study can be easily prepared in a short time. These advantages strongly demonstrate that desalination using Ag/Fe_3_O_4_ would be beneficial for the efficient treatment of radioiodine waste. To date, different silver-coated magnetic nanomaterials have been employed in a wide range of applications for specific purposes, including catalysis [42], antibacterial agents [43], imaging [44], and biosensing [45], because of their unique physical and chemical properties. This study would widen the scope of engineered nanomaterials in the field of environmental remediation. However, further optimization and validation of the process is required to investigate the industrial-scale remediation process of radioactive waste.

## 4. Conclusions

In this study, we synthesized an Ag/Fe_3_O_4_ nanoadsorbent for the desalination of radioiodine. The synthesized nanomaterials were characterized via SEM, TEM, EDS, and XRD. The composite material exhibited high adsorption capacity for I^−^ (847 mg/g) in water. The Ag/Fe_3_O_4_ composite nanoadsorbents exhibited high removal efficiency as well as ion-selective desalination in the presence of several competing ions. The material was easily recovered from the treated water by applying an external magnetic field without the significant desorption of radioactivity. Moreover, the adsorbent maintained good desalination performance during seven consecutive remediations. Consequently, it is expected that Ag/Fe_3_O_4_-based desalination will present a promising direction and can be developed as a practical method for wastewater treatment.

## Figures and Tables

**Table 1:** Calculated parameters for the adsorption isotherm fittings for the Ag/Fe_3_O_4_ nanocomposites. (*R*^2^ = coefficient of determination)

Isotherm Type	Parameters	*R* ^2^
Langmuir Model	Q_max_ = 846.860 mg/g	0.995
	K_L_ = 0.09162 L/mg	
Freundlich Model	n = 2.6	0.883
	K_F_ = 148.4 L^−1/n^ mg^(1−1/n)^	

**Table 2:** Calculated parameters for fitting the chemical kinetics of the Ag/Fe_3_O_4_ nanocomposites.

Type of Chemical Kinetics	Parameters	*R* ^2^
First-order	Q_e_ = 613 mg/g	0.976
	k_1_ = 9.0 × 10^−3^ min^−1^	
Second-order	Q_e_ = 897 mg/g	0.996
	k_2_ = 6.03 × 10^−8^ gmg^−1^min^−1^	

## Data Availability

The data presented in this article are available on request from the corresponding authors.

## Supplementary Materials

The following are available online at https://www.mdpi.com/2079-4991/11/3/588/s1, Figure S1: Figure S1. (a) Schematic route for the synthesis of Fe_3_O_4_ and Ag/Fe_3_O_4_ nanocomposites, (b) Experimental setup for the synthesis of nanoparticles and (c-e) Steps to collect Ag/Fe_3_O_4_ nanocomposites by using an external magnet; Figure S2: Figure S2. (a,b) SEM images of Fe_3_O_4_ nanoparticles, (c,d) TEM images of Fe_3_O_4_ nanoparticles, (e) Size distribution histogram of Fe_3_O_4_ nanoparticles with a standard deviation of 1.15 nm, (f) Powder XRD analysis of Fe_3_O_4_ nanoparticles.; Figure S3: Figure S3. EDS analysis of iron oxide nanoparticles; Figure S4: Figure S4. TEM images of Ag/Fe_3_O_4_ nanocomposite; Figure S5: Calibration curve to determine the unknown concentration using UV-Visible Spectrometer at 226 nm; Figure S6: (a) Pseudo-second-order kinetics study for Ag/Fe_3_O_4_, (b) Pseudo-first-order kinetics study for Ag/Fe_3_O_4_; Table. S1. Scherrer equation based crystallite size Fe_3_O_4_ nanoparticles; Table. S2. Scherrer equation based crystallite size Ag/Fe_3_O_4_ composite nanoparticles; Table. S3. Nanomaterials used for iodine removal from aqueous solutions.
