# Supplementary material for: Removal of Radioactive Iodine Using Silver/Iron Oxide Composite Nanoadsorbents
Source: Nanomaterials (Basel). 2021 Feb 26;11(3):588. doi: 10.3390/nano11030588 (PMC7996965; doi:10.3390/nano11030588)
Supplement: Supplementary file 1 [file nanomaterials-11-00588-s001.pdf]

nanomaterials

Supplementary material

Removal of Radioactive Iodine Using Silver/Iron Oxide Composite Nanoadsorbents

Mah Rukh Zia[1,†], Muhammad Asim Raza [2,3,†], Sang Hyun Park [2,3], Naseem Irfan [1], Rizwan Ahmed [1], Jung Eun Park [4], Jongho Jeon [4,*] and Sajid Mushtaq [1,2,3,*]

1 Department of Nuclear Engineering, Pakistan Institute of Engineering and Applied Sciences, P. O. Nilore, Islamabad, Pakistan; Mahrukhzia14@gmail.com (M.R.Z.); naseem@pieas.edu.pk (N.I.); Rizwanahmed@pieas.edu.pk (R.A.)

2 Advanced Radiation Technology Institute, Korea Atomic Energy Research Institute, Jeongeup 56212, Korea; m.asimraza@ust.ac.kr (M.A.R.); parksh@kaeri.re.kr (S.H.P.)

3 Radiation Science and Technology, University of Science and Technology, Daejeon 34113, Korea

4 Department of Applied Chemistry, College of Engineering, Kyungpook National University, Daegu 41566, Korea; pje1204@knu.ac.kr

* Correspondence: jeonj@knu.ac.kr (J.J.); sajidmushtaq@pieas.edu.pk (S.M.); Tel.: +82-53-950-5584 (J.J.); +92-51-9248611-3716 (S.M.)

† These Authors contributed equally to this study.

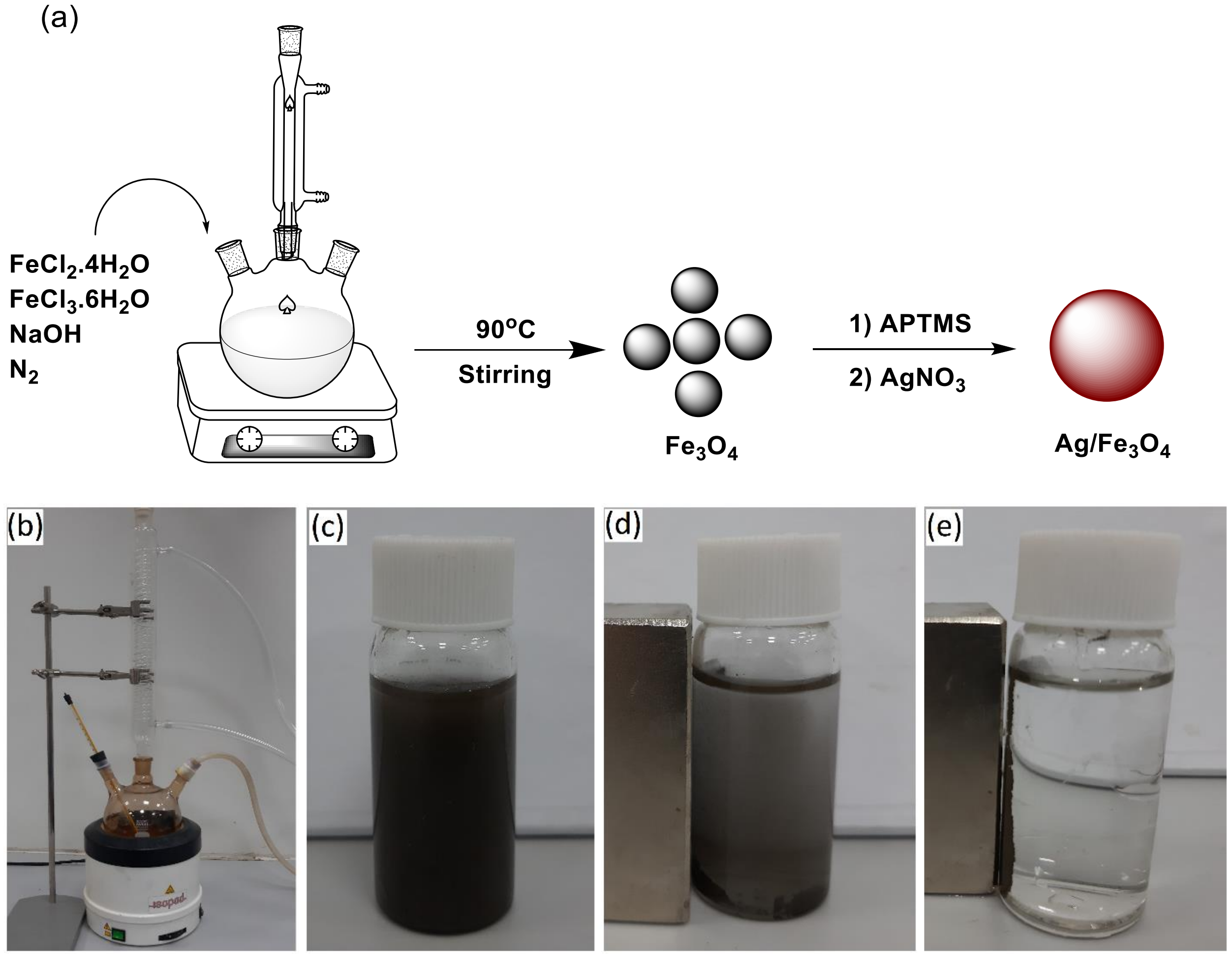

Figure S1. (**a**) Schematic route for the synthesis of Fe_3O_4 and Ag/Fe_3O_4 nanocomposites, (**b**) Experimental setup for the synthesis of nanoparticles and (**c–e**) Steps to collect Ag/Fe_3O_4 nanocomposites by using an external magnet.

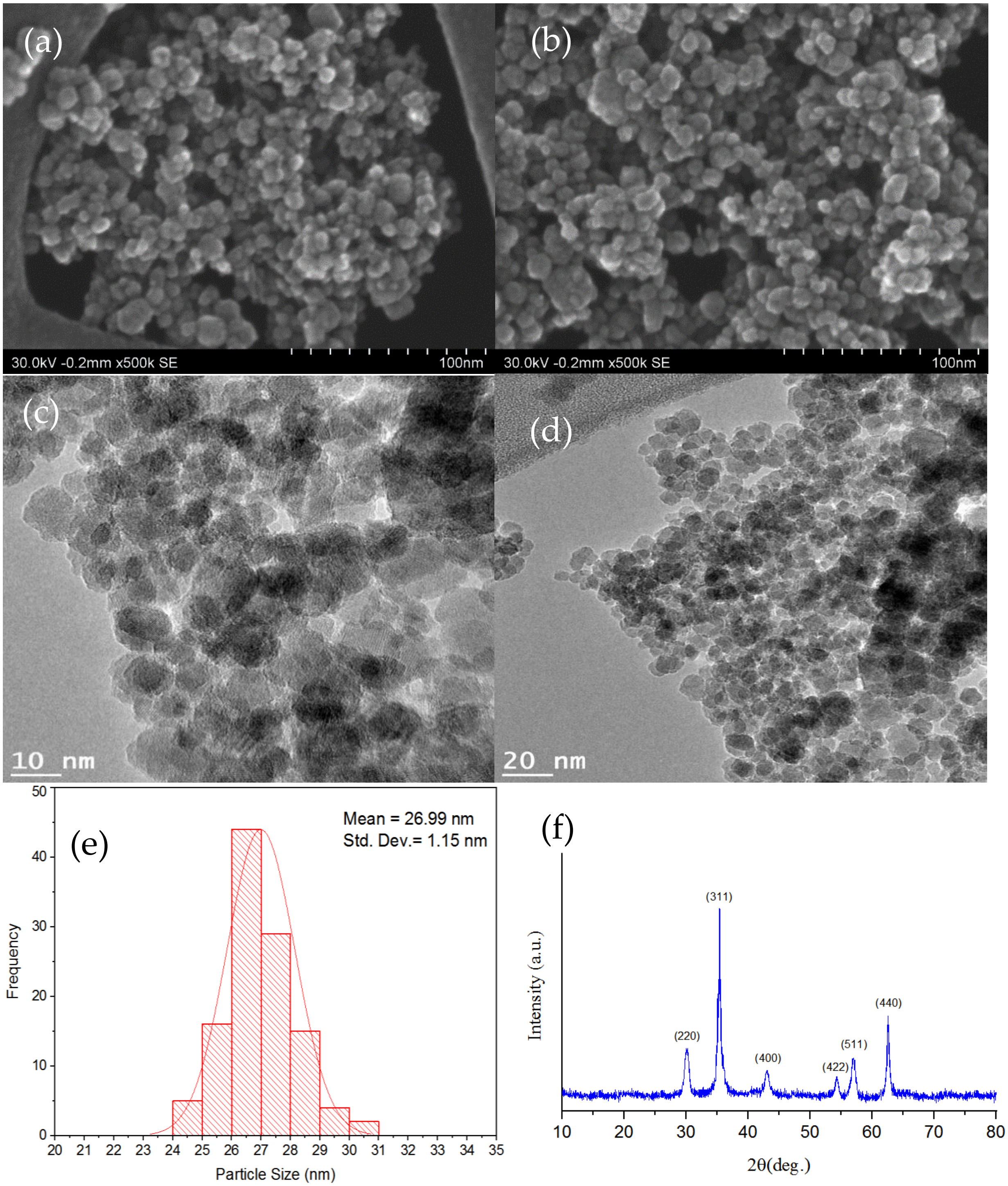

Figure S2. (**a**,**b**) SEM images of Fe_3O_4 nanoparticles, (**c**,**d**) TEM images of Fe_3O_4 nanoparticles, (**e**) Size distribution histogram of Fe_3O_4 nanoparticles with a standard deviation of 1.15 nm, (**f**) Powder XRD analysis of Fe_3O_4 nanoparticles.

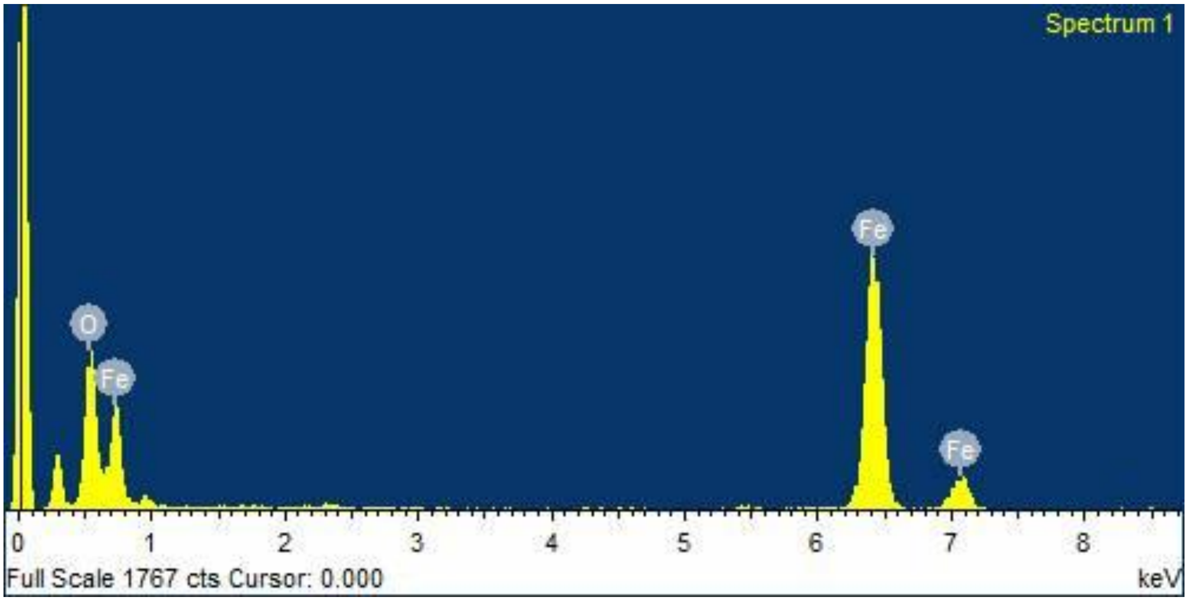

Figure S3. EDS analysis of iron oxide nanoparticles.

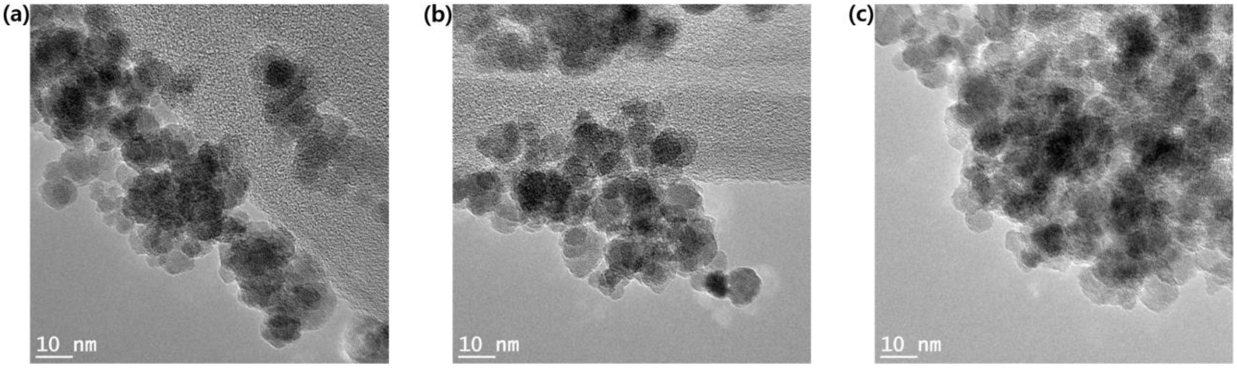

Figure S4. TEM images of Ag/Fe_3O_4 nanocomposite.

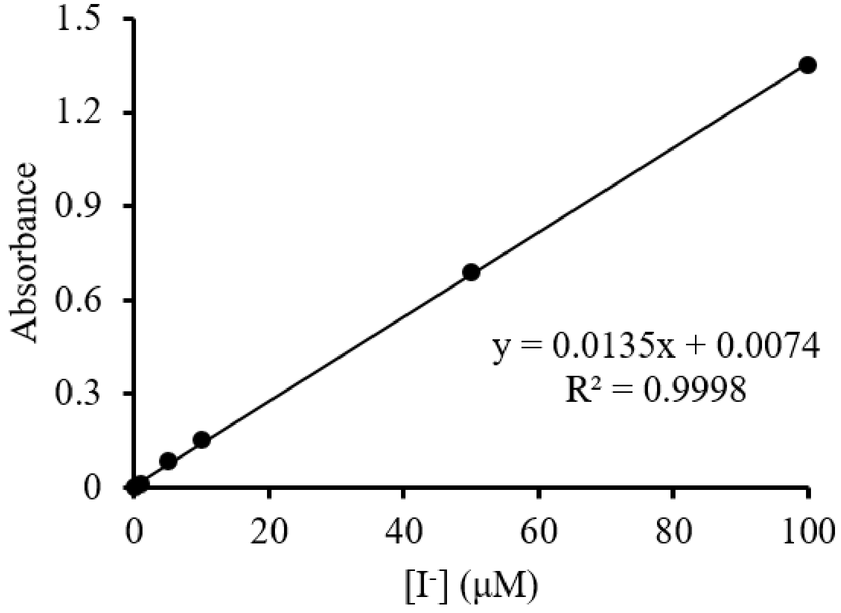

Figure S5. Calibration curve to determine the unknown concentration using UV-Visible Spectrometer at 226 nm.

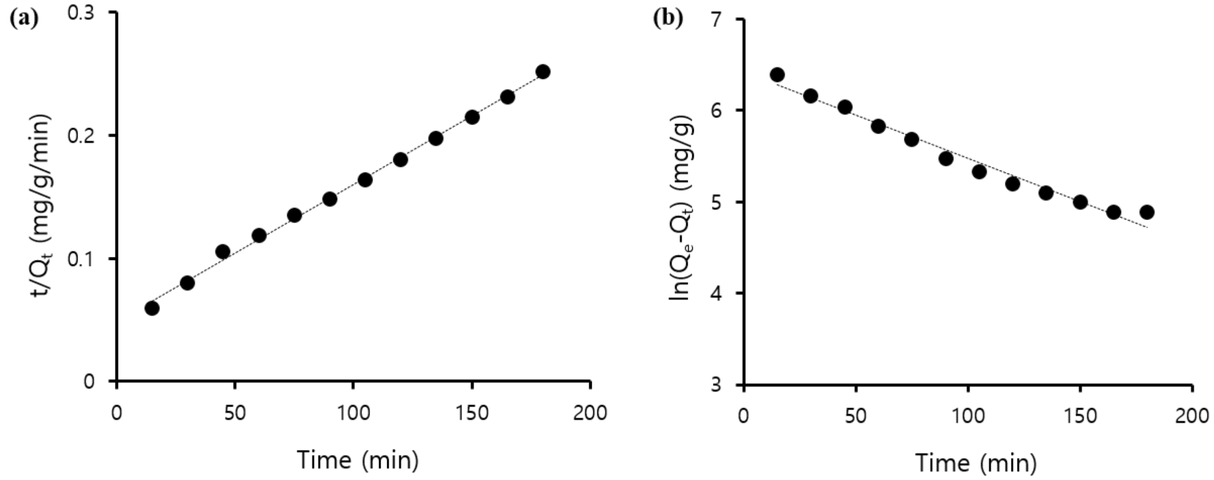

Figure S6. (**a**) Pseudo-second-order kinetics study for Ag/Fe_3O_4, (**b**) Pseudo-first-order kinetics study for Ag/Fe_3O_4.

Table S1. Scherrer equation based crystallite size Fe_3O_4 nanoparticles.

Peak position 2θ (°)	FWHM B_{size} (°)	Dp (nm)
30.10403	0.84909	10.13
35.36326	0.70679	12.33
43.03682	0.86449	9.25
54.30076	0.58961	15.83
56.99288	0.77461	12.20
62.58666	0.51349	18.92

Average Dp: 13.2 nm; Crystallite size Dp = K λ / (β cos θ); Dp: Average Crystallite size (nm); K: Scherrer constant, K = 0.94 nm; λ: X-rays wavelength λ = 1.54178 Å; β : FWHM (Full Width at Half Maximum) of XRD peak (radian); θ : XRD peak position, one half of 2θ (radian).

Table S2. Scherrer equation based crystallite size Ag/Fe_3O_4 composite nanoparticles.

Peak position 2θ (°)	FWHM B_{size} (°)	Dp (nm)	Phases
30.09569	0.52589	16.35	220, Fe_3O_4
37.88646	0.51726	16.97	111,Ag
35.60499	0.54419	16.03	311, Fe_3O_4
44.09552	0.51265	17.48	200, Ag
64.30628	0.54827	17.89	440, Fe_3O_4
77.29275	0.60798	17.49	311, Ag

Average Dp, Ag: 17.2 nm; Average Dp, Fe_3O_4: 16.7 nm.

Table S3. Nanomaterials used for iodine removal from aqueous solutions.

Nanomaterial	Target ion	Adsorption Capacity	Ref.
Bi-GO	I^- and IO_3^-	200–230 mg g^{-1}	1
Layered sodium titanate (Ag_2O-T3NT, Ag_2O-T3NF)	$^{125}I^-$	562.5 mg g^{-1a} (4.5 mmol g^{-1}) 375 mg g^{-1a} (3.0 mmol g^{-1})	2
Ag_2O@$Mg(OH)_2$	I^-	368.6 mg g^{-1a}	3
Ag_2O@NFC	I^-	650 mg g^{-1} (5.2 mmol g^{-1})	4
Fe_3O_4@PPy	I^-	1627 mg g^{-1a}	5
Nano Cu_2O-activated carbon	I^-	41.2 mg g^{-1}	6, 7
3D formicary-like δ-Bi_2O_3	I^-	255 mg g^{-1}	8
NTA-Au-CAM	I^-	24.3 mg g^{-1}	9
Silver coated iron oxide	I^-	847 mg g^{-1}	This work

Note: Ag_2O@NFC – Ag_2O nanoparticles on nanofibrillated cellulose, Fe_3O_4@PPy – magnetite nanoparticles encapsulated in the polypyrrole matrix, NTA-Au-CAM – NTA-disulfide loaded gold nanoparticles on cellulose acetate membrane, [a] Maximum monolayer adsorption capacity from Langmuir (Q_m, mg g^{-1}).

References

1. Ham, S.; Um, W.; Kim, W.-S. Development of bismuth-functionalized graphene oxide to remove radioactive iodine. *Dalton Trans.* **2019**, *48*, 478—485.
2. Yang, D.; Sarina, S.; Zhu, H.; Liu, H.; Zheng, Z.; Xie, M.; Smith, S.V.; Komarneni, S. Capture of radioactive cesium and iodide ions from water by using titanate nanofibers and nanotubes. *Angew. Chem., Int. Ed.* **2011**, *50*, 10594—10598.
3. Chen, Y.-Y.; Yu, S.-H.; Yao, Q.-Z.; Fu, S.-Q.; Zhou, G.-T. One-step synthesis of $Ag_2O@Mg(OH)_2$ nanocomposite as an efficient scavenger for iodine and uranium. *J. Colloid Interface Sci.* **2018**, *510*, 280—291.
4. Lu, Y.; Li, H.; Gao, R.; Xiao, S.; Zhang, M.; Yin, Y.; Wang, S.; Li, J.; Yang, D. Coherent-interface-assembled Ag_2O-anchored nanofibrillated cellulose porous aerogels for radioactive iodine capture. *ACS Appl. Mater. Interfaces* **2016**, *8*, 29179—29185.
5. Harijan, D.K.L.; Chandra, V.; Yoon, T.; Kim, K.S. Radioactive iodine capture and storage from water using magnetite nanoparticles encapsulated in polypyrrole. *J. Hazard. Mater.* **2018**, *344*, 576—584.
6. Zhang, X.; Gu, P.; Li, X.; Zhang, G. Efficient adsorption of radioactive iodide ion from simulated wastewater by nano Cu_2O/Cu modified activated carbon. *Chem. Eng. J.* **2017**, *322*, 129—139.
7. Zhang, X.; Gu, P.; Zhou, S.; Li, X.; Zhang, G.; Dong, L. Enhanced removal of iodide ions by nano Cu_2O/Cu modified activated carbon from simulated wastewater with improved countercurrent two-stage adsorption. *Sci. Total Environ.* **2018**, *626*, 612—620.
8. Xiong, Y.; Dang, B.; Wang, C.; Wang, H.; Zhang, S.; Sun, Q.; Xu, X. Cellulose fibers constructed convenient recyclable 3D graphene-formicary-like δ-Bi2O3 aerogels for the selective capture of iodide. *ACS Appl. Mater. Interfaces* **2017**, *9*, 20554—20560.
9. Park, J.E.; Shim, H.E.; Mushtaq, S.; Choi, Y.J.; Jeon, J. A functionalized nanocomposite adsorbent for the sequential removal of radioactive iodine and cobalt ions in aqueous media. *Korean J. Chem. Eng.* **2020**, *37*, 2209–2115.
